# TIM-3 in Leukemia; Immune Response and Beyond

**DOI:** 10.3389/fonc.2021.753677

**Published:** 2021-09-30

**Authors:** Mahnaz Rezaei, Jiaxiong Tan, Chengwu Zeng, Yangqiu Li, Mazdak Ganjalikhani-Hakemi

**Affiliations:** ^1^ Department of Immunology, Faculty of Medicine, Isfahan University of Medical Sciences, Isfahan, Iran; ^2^ Department of Hematology, First Affiliated Hospital, Jinan University, Guangzhou, China; ^3^ Institute of Hematology, School of Medicine, Key Laboratory for Regenerative Medicine of Ministry of Education, Jinan University, Guangzhou, China; ^4^ Acquired Immunodeficiency Research Center, Isfahan University of Medical Sciences, Isfahan, Iran

**Keywords:** acute lymphoblastic leukemia, acute myeloid leukemia, chronic lymphoblastic leukemia, chronic myeloid leukemia, myelodysplastic syndrome, TIM-3

## Abstract

T cell immunoglobulin and mucin domain 3 (TIM-3) expression on malignant cells has been reported in some leukemias. In myelodysplastic syndrome (MDS), increased TIM-3 expression on TH1 cells, regulatory T cells, CD8+ T cells, and hematopoietic stem cells (HSCs), which play a role in the proliferation of blasts and induction of immune escape, has been reported. In AML, several studies have reported overexpression of TIM-3 on leukemia stem cells (LSCs) but not on healthy HSCs. Overexpression of TIM-3 on exhausted CD4+ and CD8+ T cells and leukemic cells in CML, ALL, and CLL patients could be a prognostic risk factor for poor therapeutic response and relapse in patients. Currently, several TIM-3 inhibitors are used in clinical trials for leukemias, and some have shown encouraging response rates for MDS and AML treatment. For AML immunotherapy, blockade TIM-3 may have dual effects: directly inhibiting AML cell proliferation and restoring T cell function. However, blockade of PD-1 and TIM-3 fails to restore the function of exhausted CD8+ T cells in the early clinical stages of CLL, indicating that the effects of TIM-3 blockade may be different in AML and other leukemias. Thus, further studies are required to evaluate the efficacy of TIM-3 inhibitors in different types and stages of leukemia. In this review, we summarize the biological functions of TIM-3 and its contribution as it relates to leukemias. We also discuss the effects of TIM-3 blockade in hematological malignancies and clinical trials of TIM-3 for leukemia therapy.

## 1. An Introduction of T Cell Immunoglobulin and Mucin Domain-3

T cell immunoglobulin and mucin domain 3 (TIM-3) is a cell surface molecule which was first identified approximately two decades ago on terminally differentiated CD4+ type 1 helper T cells (TH1 cells) and CD8+ cytotoxic T cells (CTLs) (1). Later, its expression was observed on other T cell subtypes, excluding TH2 cells, as well as some other immune cells including dendritic cells (DCs), natural killer (NK) cells, monocytes, macrophages, and mast cells (2–4). Also in some cancers, the malignant cells can express TIM-3 (5–8).

The gene that encodes TIM-3 is *havcr2* (hepatitis A virus cellular receptor 2), which is located on chromosome 5q33.2. Due to the genes encoded in this region, including interleukin (IL)-4 and IL-5, this locus is known to be linked to allergies and autoimmune disease (9, 10).

TIM-3 is a single transmembrane (TM) molecule whose extracellular tail contains a N-terminal IgV domain. This domain is subsequently followed by a mucin domain with glycosylation sites, which explains where its name comes from. After the mucin domain, there is a link peptide with N-linked glycosylation sites and then the TM domain followed by the cytoplasmic tail in the C-terminus (2, 11).

**Figure 1:**
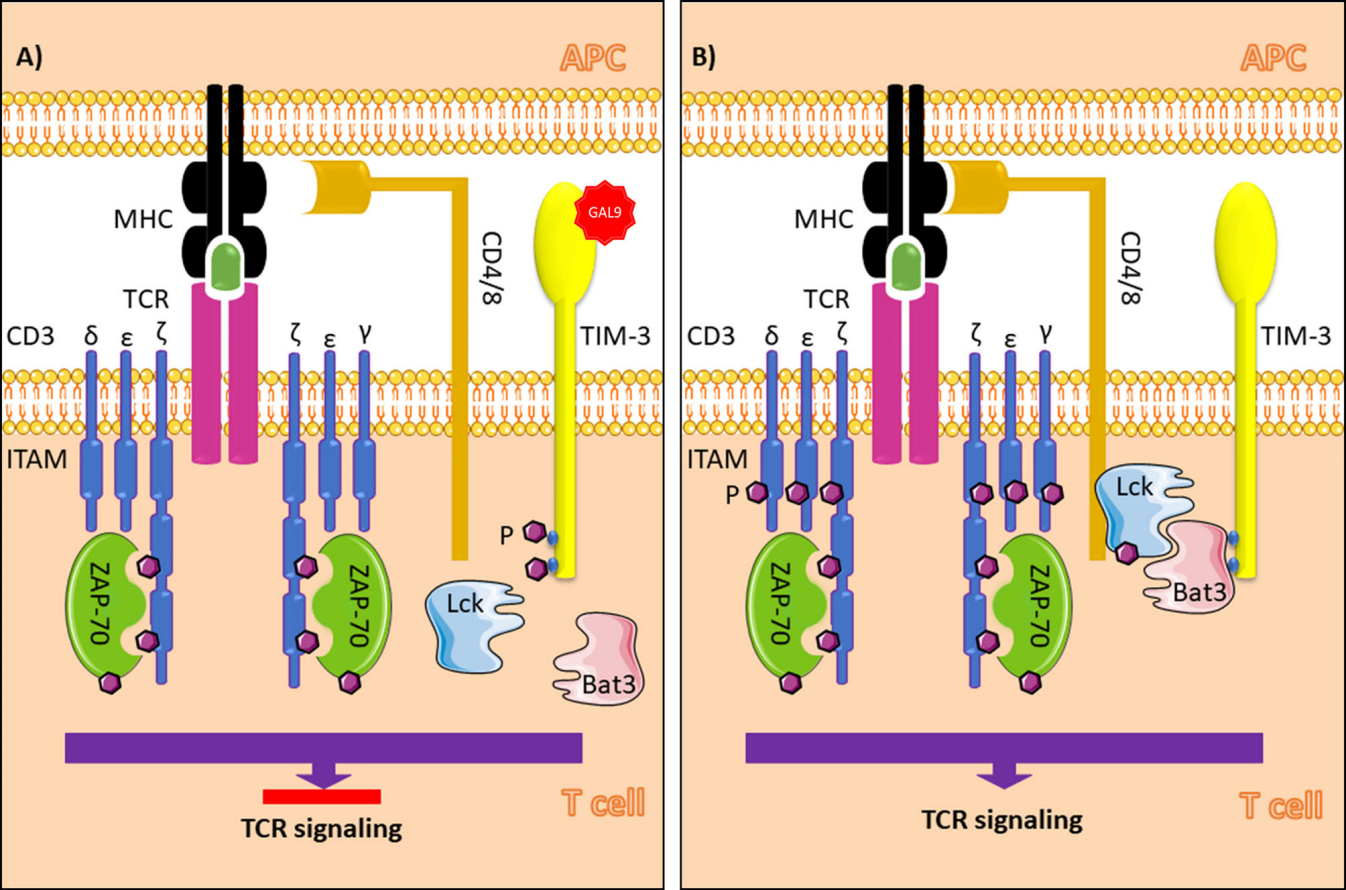
TIM-3 signaling in T cells in the presence **(A)** and absence **(B)** of galectin-9. In the absence of Gal-9, Bat3 binds to tyrosine residues in the cytoplasmic tail of TIM-3 (Y256 and Y263 in mice and Y26 and Y272 humans). This process leads to accumulation of the active form of Lck, which promotes phosphorylation of Zap70 and T cell signaling when the MHC peptide-TCR complex is formed. In the presence of Gal-9, as the ligand for TIM-3, phosphorylation of the tyrosine residues results in Bat3 release from the cytoplasmic tail. In this paradigm, Bat3 cannot form a complex with Lck; thus, TIM-3 induction inhibits T cell signaling. TIM-3, T cell immunoglobulin and mucin domain-3; Gal-9, galectin-9; Bat3, HLA-B associated transcript 3; TCR, T cell receptor; ITAM, immunoreceptor tyrosine-based activation motif; APC, antigen presenting cell; MHC, major histocompatibility complex; Ag, antigen. The figure was produced with the assistance of Servier Medical Art (https://smart.servier.com).

TIM-3 does not contain classic inhibitory tyrosine-based motifs such as ITIM (immunoreceptor tyrosine-based inhibition) or ITSM (immunoreceptor tyrosine-based switch). However, there is a conserved region with five tyrosine residues, which two of them, Y265 and Y272 in humans (Y256 and Y263 in mice), are assumed to be phosphorylated after the interaction of TIM-3 with its ligands. Itk, a Tec family tyrosine kinase, and Fyn and Lck, two Src family kinase members, are known to be involved in the TIM-3 signaling pathway. A study of Jurkat T cells revealed that Tim-3 overexpression following T cell receptor and CD-28 induction promotes Lck or Fyn-dependent phosphorylation of Y256 and Y263. This event leads to accumulation of proteins with SH2 domains, such as the p85 subunit of phosphoinositide 3-kinase (PI3K) and phospholipase C-γ1 (PLC-γ1), to the cytoplasmic tail of TIM-3. Furthermore, TIM-3 activation enhances NFAT and NF-κB activation thorough interaction with ZAP-70 and SLP-76, components of the TCR signaling pathway. In contrast, another study of Jurkat cells demonstrated that TCR induction in TIM-3-expressing cells suppresses AP-1 and NFAT activation, resulting in impaired IL-2 production (12–14) (**Figure 1**). This discrepancy can likely be explained by differences in acute *vs*. chronic TCR induction and whether TIM-3 expression is secondary to TCR induction or vice versa. In addition, HLA-B associated transcript 3 (Bat3) can directly bind to the cytoplasmic tail of TIM-3 and prevent signal induction in the absence of TIM-3 ligands (15).

There are four known ligands for TIM-3. The first and most famous ligand is galectin-9 (Gal-9), which has been reported to induce apoptosis in TH1 cells. Gal-9 can also bind TIM-3 on other cells under other circumstances and result in activation of different pathways (16, 17). High-mobility group protein B1 (HMGB1) is a type of damage associated molecular pattern (DAMP), known as “alarmin”, which is released from damaged cells and induces the activation of phagocytes. HMGB-1 can bind TIM-3 in different contexts and does not always lead to one outcome (18). Another TIM-3 ligand is a well-known “eat-me” signal induction molecule phosphatidylserine (PtdSer). Appearance of PtdSer on the outer surface of cell membranes leads to phagocytosis. The same process is promoted when PtdSer interacts with TIM-3 on myeloid cells, but it does not always induce a same inflammatory response (19). The last ligand is carcinoembryonic antigen cell adhesion molecule 1 (CEACAM-1), which can have both *cis* and *trans* interactions with TIM-3 (20).

## 2. Alterations in TIM-3 Expression in Patients With Leukemia

### 2.1. TIM-3 in Myeloid Leukemias

#### 2.1.1. Myelodysplastic Syndrome (MDS)

Myelodysplastic syndrome (MDS) is a heterogenous hematologic disorder, and its clinical manifestations include a wide spectrum ranging from mild and single lineage cytopenia to high-risk MDS, which rapidly progresses to acute myeloid leukemia (AML) (21–24).

Several studies of T cell populations in MDS patients have been performed so far. Ozkazanc et al. observed a subpopulation of CD4+ T cells in the bone marrow aspirate of MDS patients expressing the exhaustion markers programmed cell death 1 (PD-1), TIM-3, and lymphocyte activation gene 3 (LAG3) (25). Fu et al. classified CD4+ T cells in MDS patients and reported increased TIM-3 expression on type 1 helper T cells and regulatory T cells (26). In another study, Tao et al. reported that in addition to a decrease in the population of CD8+ T cells in MDS patients, these cells express high levels of TIM-3, and as expected, TIM-3+ CD8+ T cells express higher perforin and granzyme B and lower CD95 (also known as Fas) compared to TIM-3- CD8+ T cells in MDS patients (27). In a recent study, Tao et al. reported increased expression of Gal-9 on myeloid derived suppressor cells (MDSCs) in MDS patients and theorized that the interaction between the Gal-9 on MDSC cells and TIM-3 induces T cell exhaustion in MDS patients (28). All of these studies have suggested the use of immune checkpoint inhibition to restore immune surveillance in MDS patients.

Although different TIM-3 localization patterns (intracellular and extracellular expression) have been reported in different MDS cell lines, they all agree on increased overall expression of TIM-3 on hematopoietic stem cells (HSCs; CD34+ CD38- Lin-) in MDS patients compared to control groups (29, 30). Tao et al. reported that TIM-3 expression on HSCs in MDS patients is increased compared to healthy individuals and is close to the expression level in AML patients. Their study on TIM-3+ HSCs revealed decreased expression of differentiation related molecules, increased proliferation, and decreased apoptosis, compared to TIM-3- HSCs. This group also reported a correlation between a higher percentage of TIM-3 expression on HSCs in MDS patients and an increased WPSS score, greater than one cytopenia lineage, higher blast counts in bone marrow smears, and worse karyotype (21). Because these characteristics are known as indicators of disease progression, Tao et al. suggest that a higher percentage of TIM-3 reflects higher risk for MDS transformation to leukemia. Tcvetkov et al. have also reported increased levels of TIM-3 and its ligand, Gal-9 on bone marrow cells from MDS patients (31). In this regard, Asayama et al. claimed that the bone marrow microenvironment and Gal-9/TIM-3 axis in HSCs not only leads to MDS formation but is also related to disease progression to leukemia in MDS patients. This group also reported elevated levels of Gal-9 in MDS patients, highlighting the TIM-3/Gal-9 axis role in the proliferation of blasts and induction of immune escape, supporting disease progression (29) (**Table 1**). It is not clear that TIM-3 expression on HSCs is weather an early trigger for MDS formation and HSCs’ possible transformation to LSCs, or it is a secondary event to MDS formation and acts as an accelerator for disease progression. Hence, more studies are needed.

**Table 1:** TIM-3 alteration in leukemias and reported correlations.

Leukemia	Alteration	Reported Correlation
**MDS**	TIM-3 overexpression on CD4+ T cells	No published evidence
	TIM-3 overexpression on CD8+ T cells	No published evidence
	TIM-3 overexpression on HSCs	Increase in WPSS score More than one lineage cytopenia Higher blast count in BM smear Higher risk for MDS transformation to leukemia
**AML**	TIM-3 overexpression on LSCs	Response to chemotherapy (?)
	TIM-3 overexpression on CD4+ T cells	Higher in patients with FLT3-ITD mutation
	TIM-3 overexpression on CD8+ T cells	Higher in high-risk AML patients
	Increase in TIM-3+ PD-1+ T cells	Higher chance for leukemia relapse
	TIM-3 overexpression on regulatory T cells	Poor prognosis in patients with normal cytogenetics
	TIM-3 upregulation on NK cells	Better clinical outcome
**CML**	Increase in proportion of PD-1+ TIM-3- CD8+ T cells	Poor response to TKI therapy
**B-ALL**	Overexpression of TIM-3 and PD-1 on T cells	Leukemia Relapse after allogenic HSCT (?)
	Overexpression of TIM-3 on T cells	Prognostic risk factor for B-ALL relapse
	Overexpression of TIM-3 in BM and PBMC	No published evidence
**T-ALL**	Expression of TIM-3 in leukemic cells	Positively related to chemoresistance
	Overexpression of TIM-3 in BM and PBMC	No published evidence
**CLL**	Increase in percentage and absolute count of TIM-3+ T cells	Positive correlation with advanced clinical stage
	Overexpression of TIM-3 on NK cells of PB	Poor prognostic factor

MDS, myelodysplastic syndrome; TIM-3, T cell immunoglobulin and mucin domain-3; CD, cluster of differentiation; WPSS, WHO (world health organization) classification-based prognostic scoring system; HSC, hematopoietic stem cell; BM, bone marrow; AML, acute myeloid leukemia; LSC, leukemia stem cell; FLT3-ITD, internal tandem duplications in the FLT3 tyrosine kinase; PD-1; progmammed cell death protein-1; NK cell, natural killer cell; CML, chronic myeloid leukemia; TKI, tyrosine kinase inhibitors; B-ALL, acute lymphoblastic B-precursor leukemia; HSCT, hematopoietic stem cell transplantation; PBMC, peripheral blood mononuclear cell; T-ALL, T-cell acute lymphoblastic leukemia; CLL, chronic lymphoblastic leukemia; PB, peripheral blood.

#### 2.1.2. Acute Myeloid Leukemia (AML)

Acute myeloid leukemia (AML) is a progressive myeloproliferative malignancy with low overall survival (32). AML has a heterogenous genetical nature; while about 45% of patients may display normal karyotype, others may show molecular mutations including internal tandem duplications in the FLT3 tyrosine kinase (FLT3-ITD). In some cases, cytogenetic abnormalities have also been reported. All of these abnormal alterations are defined with different prognostic values (33–37). Studies on the expression of TIM-3 in AML can be divided into two types: expression of TIM-3 on immune cells, particularly T cells and NK cells, and expression of TIM-3 on LSCs. Since genetic abnormalities in AML can be considered as prognostic risk factors, there may be correlations between them and the overexpression of TIM-3 in leukemia cells and/or immune cells in AML; hence more studies are required in this aspect.

When it comes to leukemia, there is a distinct group of stem cells (SCs) called leukemic stem cells (LSCs), which have different molecular patterns, sometimes including cytogenetic abnormalities and mutations, higher proliferation rates, and resistance to apoptosis, and they reproduce to form the malignant cell population (32, 38). One of the distinguishing characteristics of LSCs in AML is their upregulated TIM-3 expression. Many studies have reported overexpression of TIM-3 on LSCs but not on healthy hematopoietic stem cells (HSCs) (CD34+ CD38– Lin-) except in acute promyelocytic leukemia (M3) (8, 30, 39–41). Therefore, targeting TIM-3 with an antibody or microRNAs leads to LSC eradication with no effects on normal HSCs (8, 42–44). In addition, Jan et al. suggested that TIM-3 could be used to separate leukemic and healthy SCs (39).

Later, several studies have focused on the role of TIM-3 in leukemic cells in AML. It has been revealed that there is an autocrine stimulatory loop in AML cells that works through the interaction between TIM-3 with Gal-9, which leads to phosphorylation of ERK (extracellular signal-regulated kinase) and protein kinase B (PKB, also known as AKT). This process results in induction of the β-catenin pathway and nuclear factor kappa-light-chain-enhancer of activated B cells (NF-κB) activation, which is important for cell survival and disease progression, which explains why targeting TIM-3 on these cells promotes apoptosis (45, 46). Moreover, ligation of TIM-3 and Gal-9 in AML cell lines leads to phosphatidylinositol-3 kinase (PI-3K)/mammalian target of rapamycin (mTOR) pathway and ERK pathway activation, resulting in hypoxia-inducible factor 1-alpha (HIF-1α), vascular endothelial growth factor (VEGF), and TNF-α production (47).

TIM-3 is also involved in immune evasion in AML. Folgiero et al. ascertained that indoleamine 2,3-dioxygenase1 (IDO1), which is known as an anti-inflammatory enzyme (48), can be produced by AML blasts in response to TIM-3/Gal-9-dependent interferon gamma (IFN-γ) production from natural killer (NK) cells (49). Furthermore, Goncalves Silva et al. reported that increased amounts of TIM-3 in plasma, probably due to its secretion from AML blasts, and the soluble form of TIM-3 (sTIM-3), which is formed by its shedding from the surface of AML blasts, can inhibit the release of interleukin-2 (IL-2), a vital cytokine involved in the activation and function of T cells and NK cells, from immune cells (50).

Reports of the influence of TIM-3 on chemotherapeutic agents are controversial. Higher TIM-3 expression on AML blasts has been reported to be an enhancer for better response to chemotherapy by Xu et al. (51); however, Dama et al. noted that this overexpression is positively correlated with chemotherapy failure (52). It is evident that, more studies are required to address this controversy.

As mentioned above, upregulation of TIM-3 expression on immune cells leads to their dysfunctional activity in AML. Several studies have reported upregulation of TIM-3 on CD4+ and CD8+ T cells (25, 53–57). Ozkazanc et al. reported that the exhausted phenotype of CD4+ T cells, which is characterized by the expression of TIM-3, LAG-3, and PD-1, in AML patients is induced by the co-stimulatory signals of AML blasts (25).

In contrast, some studies have suggested meaningful associations between TIM-3 overexpression levels and prognosis. Li et al. revealed that TIM-3 overexpression on CD4+ T cells in AML patients that have an FLT3-ITD mutation, a poor prognostic factor, was higher than that in those who did not have this mutation (53). This group also reported higher levels of TIM-3 expression on CD8+ T cells in high-risk AML patients compared to low-risk patients. Kong et al. noted that for patients with elevated Tim3+ PD-1+ T cells after allogenic stem cell transplantation, leukemia relapse is more predictable (55). Later, Zahran et al. determined a positive correlation between TIM-3 expression and poor prognosis in AML patients with normal cytogenetics (56). Tan et al. demonstrated that higher TIM-3+ CD244+ CD8+ T cells are observed in M4 AML patients compared to M3 patients. This group also reported that AML patients have decreased TIM-3+ T cell portions by the time they are in the complete remission phase (57).

In a remarkable recent study, Rakova et al. observed NK cell activity in AML patients. This group revealed that TIM-3 upregulation on these cells was associated with increased activity of NK cells and resulted in better clinical outcome for AML patients (58) (**Table 1**). As noted, exceptional studies have been performed on TIM-3 in AML, yet so many aspects are still intact or not completely examined. For instance, how does TIM-3 affect the function of other immune cells, are there any other links between TIM-3 overexpression on different immune cells and different aspects of prognosis (i.e., resistance to different therapies, AML relapse, overall survival, refractory AML), are there mechanisms underlying upregulation of TIM-3 on LSCs, and how overexpression changes different aspects of function and stability of LSCs?

#### 2.1.3. Chronic Myeloid Leukemia (CML)

Reciprocal translocation between chromosome 9 and 22 [t(9,22)] creates the oncokinase BCR-ABL1, developing a myeloproliferative hematologic malignancy called chronic myeloid leukemia (CML). Tyrosine kinase inhibitors (TKIs) are considered first-line, approved treatments for CML patients that direct a deep molecular response hampering leukemia progression (59, 60).

Many studies reported impaired anti-tumor immunity in CML patients. Among them, Bruck et al. (61) observed TIM-3 overexpression on exhausted CD4+ and CD8+ T cells in untreated CML patients. This is not an unexpected report, since TIM-3 is known as an exhaustion marker in T cells. However, they also reported a correlation between PD-1+ TIM-3- CD8+ T cells and poor response to TKIs (**Table 1**). This may seem surprising, because we are more used to announce TIM-3 as a marker for disease progression, therapy resistance, probable relapse and more complicated situation for treatment. Unfortunately, there is not enough reports about TIM-3 in CML. TIM-3 and response to TKIs in CML is not well studied and more specified and detailed studies are required to clarify its possible role in better prognosis.

Dysfunctional immunity plays a major role in malignancy formation. Moreover, possibility of CML relapse due to the existence of undetectable residual leukemia stem cell, even after successful TKI therapy, magnifies the critical role of functional immune system. Therefore, much more clinical studies are required to examine the expression of TIM-3 in other immune cell types in CML as well and establish its role in formation, therapy resistance, relapse of CML, and immune scoring in this malignancy. Also, the possibility of the expression of TIM-3 on leukemic cells should not be ignored.

### 2.2. TIM-3 in Lymphoblastic Leukemias

#### 2.2.1. Acute Lymphoblastic Leukemia (ALL)

Acute lymphoblastic B-precursor leukemia (B-ALL) is the most common hematologic malignancy in children, which is formed by uncontrolled proliferation and a defect in lymphoid progenitors (62, 63). A few studies have been performed to determine the role of TIM-3 in B-ALL relapse, which is a challenging issue in this subtype. Although Liu et al. observed T cell exhaustion, characterized by TIM-3 and PD-1 expression, in post-allogenic-HSCT B-ALL relapse, they could not describe a more detailed relationship between increased immune checkpoint expression and relapse (64). Later in 2020 in a study on pediatric B-ALL, Blaeschke et al. reported overexpression of TIM-3 alone and in combination with PD-1 on CD4+ T cells in the bone marrow with no significant difference in T cell subpopulations, and defined TIM-3 as a substantial prognostic risk factor for relapse in B-ALL patients (65). This group also claimed that CD200 may be responsible for TIM-3 induction on T cells in ALL. These findings have not yet been shown *in vivo*.

While ALL is considered as the most common malignancy in children, about 15% of cases are T-cell acute lymphoblastic leukemia (known as T-ALL), in which almost 20% of them do not survive. According to records, this malignancy is not that common in adults (25% of all ALL cases), but mortality rate is higher than pediatric patients (about 50%) (66, 67).

Horlad et al. demonstrated that TIM-3 expression in leukemic cells is possibly related to resistance to chemotherapy in T-ALL patients (68), noting that more studies are needed. In a more recent study, Balajam et al. included both B-ALL and T-ALL cases and documented TIM-3 overexpression in bone marrow and peripheral blood mononuclear cells (PBMCs) in ALL patients compared to control individuals (69) (**Table 1**). Thus, more studies regarding the possible diagnostic and/or prognostic value of TIM-3 in ALL are recommended.

Chronic lymphocytic leukemia (CLL) is defined as a hematologic malignancy in which CD5+ B cells aggregate not only in peripheral blood but also in bone marrow and secondary lymphoid organs (70, 71). CLL has different contribution patterns all over the world (72, 73) and displays a wide spectrum of clinical manifestations ranging from asymptomatic individuals to fast progressing malignant cases (71, 74). Poor response to common treatments, refractory CLL, and leukemia relapse after a period of remission are other challenging issues in these patients (75).

Two major studies have been performed regarding the immunologic exhaustion profile in CLL patients. Allahmoradi et al. examined CD4+ T cells and Taghiloo et al. explored CD8+ T cells in CLL (76). Both studies reported that exhausted CD4+ and CD8+ T cells, characterized by PD-1 and TIM-3 expression, have a higher percentage and absolute count in CLL patients compared with healthy individuals. The same result was observed when only TIM-3+ T cells were considered. Moreover, lower proliferation and protective cytokine production by these TIM-3+ T cells has been reported. Furthermore, both Allahmoradi et al. and Taghiloo et al. claimed that the percentage and absolute count of TIM-3+ PD-1+ CD4+ T cells and TIM-3+ PD-1+ CD8+ T cells were positively correlated with advanced clinical stages for CLL patients. Later, Hadadi et al. reported TIM-3 overexpression in another immune cell type, called natural killer (NK) cells, which plays a critical role in eliminating malignant cells, in the peripheral blood of CLL patients. This study reported that an upregulated TIM-3 profile is a linked to poor prognostic factors for CLL patients (77) (**Table 1**). Together, these three studies have presented TIM-3 as a “promising biomarker and possible target for future immunotherapy” (76, 77). More detailed clinical studies are required to clarify the possible role of TIM-3 in CLL pathogenesis and its association with cases of leukemia relapse, response to standard therapeutic agents and molecular prognostic factors of CLL.

## 3. Effect of TIM-3 Blockade in Leukemia

TIM-3 blockade alone was demonstrated to have anti-solid tumor effects in preclinical studies by improving the *ex vivo* proliferation of tumor-infiltrating T cells and increasing the secretion of the cytokines IFN-γ and TNF-α (78, 79). Other checkpoint inhibitors, such as the PD-1/PD-L1 axis and LAG-3, combined with TIM-3 blockade could further enhance the immune function of tumor-infiltrating T cells (78, 79). In clinical trials, TIM-3 blockade, especially in combination with PD-1 blockade, has demonstrated reliable preliminary results against solid tumors (79–81). Based on the expression characteristics of TIM-3, which is expressed both on AML cells and exhausted T cells (52, 57), TIM-3 blockade may have dual effects: directly inhibiting AML cell proliferation and revising the exhausted T cell phenotype and restoring T cell function. Moreover, overexpression of TIM-3/Gal-9 has been found in AML patients who failed chemotherapy, suggesting that targeting TIM-3/Gal-9 in combination with chemotherapy induction may be an alternative approach to increasing the complete remission rate of patients with AML (52, 82). The results of Kikushige Y et al., who found that injection of TIM-3+ AML cells in immune-deficient mice could establish an AML model and subsequent TIM-3 blockers could alleviate disease, supported the efficacy of this targeted approach (8). In AML allogeneic hematopoietic stem cell transplantation (allo-HSCT) models, TIM-3 blockade may lead to activation of macrophages to eradicate AML stem cells and ameliorate disease (39, 83). Recently, an *in vitro* study demonstrated that bispecific and split CAR T cells (BissCAR-T cells) that target CD13 and TIM-3 can specifically eliminate AML cells (84). Because there are no known life-essential cells that express both CD13 and TIM-3, BissCAR-T cells can selectively kill AML cells while reducing toxicity to human hematopoietic stem cells and other normal tissues. In addition, anti-human TIM-3 was identified as a potential strategy for curing AML by targeting LSCs (84).

TIM-3 is highly expressed in peripheral blood and bone marrow exhausted T cells in a variety of hematological malignancies, including acute lymphoblastic leukemia (ALL), chronic lymphocytic leukemia (CLL), and multiple myeloma (MM) (57, 77, 85). However, few reports have demonstrated the significant effects of TIM-3 inhibitors alone in the above diseases, and the reason may be due to the fact that TIM-3 suppression can partially restore T cell activation but it is unable to overcome the T cell exhausted status. Moreover, there is high expression of a number of immune checkpoint proteins, such as PD-1 and TIGIT, in T cells from patients. For example, blockade of PD-1 and TIM-3 failed to restore the function of exhausted CD8+ T cells in the early clinical stages of CLL (85), indicating the effects of TIM-3 blockade may be different in AML and other leukemias, further studies are required to evaluate the efficacy of TIM-3 inhibitors in different types and stages of leukemia as well as in different leukemia bone marrow microenvironments.

## 4. TIM-3 Inhibitors for Leukemia Therapy in Clinical Trials

**Figure 2:**
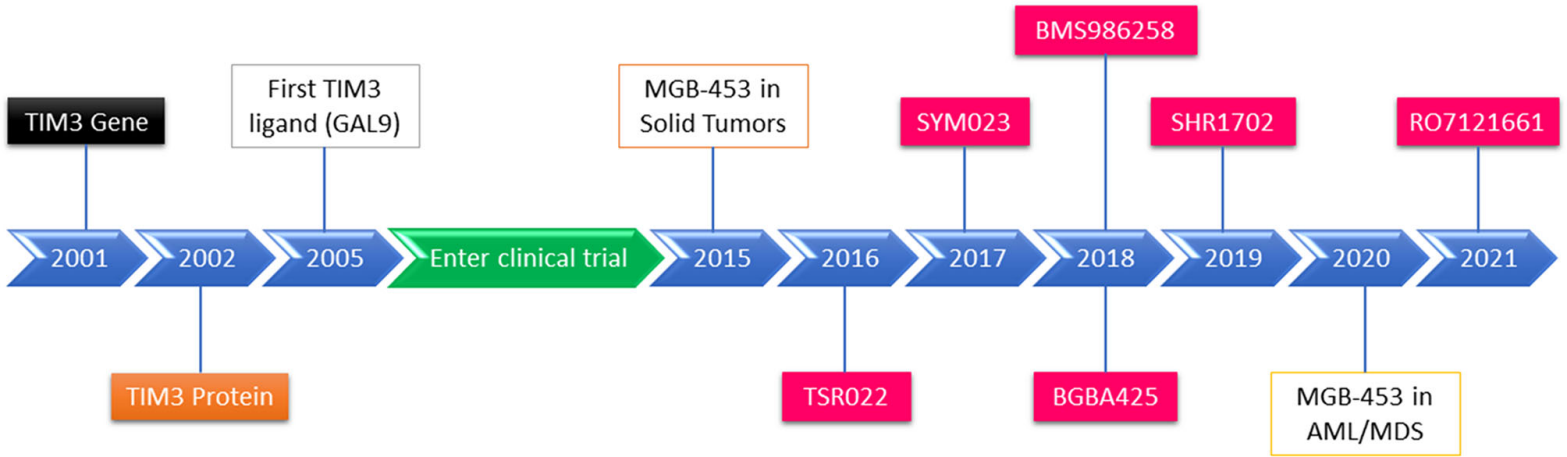
Schematic diagram of TIM-3 identification, TIM-3 inhibitor development, and TIM-3 blockade in cancer and leukemia therapies in clinical trials.

Currently, the TIM-3 inhibitors used in clinical trials include MBG453 (also known as Sabatolimab), TSR-022, BMS-986258, LY3321367, SYM023, BGB-A425, and SHR-1702 (86, 87). However, MBG453 and SHR-1702 have begun to be used in clinical trials for leukemia immunotherapy only (86) (**Table 2** and **Figure 2**).

**Table 2:** Anti–TIM-3 agents and associated clinical trials in leukemia.

Clinical trial identifier	Phase	Start date		Status	Cancer type (population, N)	Interventions and Combination		Primary Outcome Measures	Secondary Outcome Measures
NCT03066648 (88)	Ib	July 6, 2017		Active, not recruiting	ND or R/R AML, HR-MDS (N≈243)	MBG453(alone)		Safety, DLTs	PK, ORR, etc.
						+HMA			
						+Anti-PD-1			
						+HMA+ Anti-PD-1			
NCT03940352	Ib	June 24, 2019		Recruiting	AML, HR-MDS (N≈80)	MBG453 +HDM201		Safety, DLTs	ORR, BOR, etc.
						Venetoclax+HDM201			
NCT04443751 (86)	I	September 10, 2020		Recruiting	R/R-AML, HR-MDS(N≈42)	SHR-1702		MTD,RP2D	Safety, PK, etc.
NCT03946670 (89)	II	June 4, 2019		Active, not recruiting	HR-MDS (N≈127)	MBG453+HMA		CR rate, PFS	OS, EFS, etc.
						Placebo+HMA			
NCT04150029 (89)	II	September 1, 2020		recruiting	ND AML (N≈86)	MBG453+HMA+Venetoclax		Safety, DLTs CR rate	CR/CRi rate, OS, etc.
NCT04823624	II	September 2021		Not yet recruiting	LD-MDS (N≈20)	MBG453(alone)		ORR	OS, PFS, etc.
NCT04623216	Ib/II		July 22, 2021	recruiting	AML (MRD+/post-aHSCT) (N≈59)	Part 1	MBG453 400mg Q4W	DLTs, R/R rate	III or IV aGVHD rate, etc.
							MBG453 800mg Q4W		
						Part 2	MBG453(age>18)		
							MBG453(12>age>18)		
NCT04878432	II		June 30, 2021	Not yet recruiting	HR-MDS (N≈90)	MBG453 + HMA or INQOVI (oral decitabine))		Safety	CR, mCR, etc.
NCT04812548	II		May 24, 2021	Not yet recruiting	HR-MDS (N≈76)	MBG453+HMA+Venetoclax		Safety, DLTs, CR rate	ORR, PFS, etc.
NCT04266301	III		June 8, 2020	recruiting	HR-MDS, CMML-2 (N≈500)	MBG453+ Azacitidine		OS	Safety, CR, etc.
						Placebo+ Azacitidine			
CTR20201781	III		August 6, 2020	recruiting	HR-MDS, CMML-2(N≈100)	MBG453+Azacitidine		OS	Safety, CR, etc.
						Placebo+Azacitidine			

AML, acute myeloid leukemia; aGVHD, acute graft versus host disease; BOR, best overall response; CMML-2, chronic myelomonocytic leukemia-2; CR, complete response; CRi, complete remission with incomplete hematologic recovery; DLTs, dose-limiting toxicities; EFS, event-free survival; HR-MDS, higher-risk myelodysplastic syndrome; HMA, hypomethylating agent, LD-MDS, lower risk MDS; mCR, marrow remission; MTD, maximum tolerated dose; ND, newly diagnosed; ORR, overall response rate; OS, overall survival; PD-1, programmed cell death 1; PFS, progression-free survival; PK, pharmacokinetic; R/R, relapsed/refractory; RP2D, recommended phase 2 dose; TIM-3, T-cell immunoglobulin domain and mucin domain 3.

Although a number of TIM-3 blockade clinical trials for malignant tumor have been reported, MBG453 is the only inhibitor that has shown preliminary efficacy and safety in clinical studies for MDS and AML (86). Currently, eight phase I/II clinical trials are ongoing for AML and MDS with MBG453 monotherapy or the combination of different agents such as hypomethylating agents (HMAs), PD-1 inhibitors, HDM201 (an MDM2 inhibitor), and venetoclax. There is also just one phase I trial administering SHR-1702 for relapsed/refractory (R/R) AML and higher-risk myelodysplastic syndromes (HR-MDS). Moreover, there are two phase III trials of MBG453 + azacitidine for HR-MDS and chronic myelomonocytic leukemia-2 (CMML-2) (**Table 2**).

Most phase I/II trials of TIM-3 inhibitors for AML or MDS have been initiated in the past two years, and the trials are still ongoing; thus, the final results have yet to be released. Currently, positive results from several trials have indicated that the ORR of 69 patients with HR-MDS or AML who received MBG453 plus decitabine (Dec) (cutoff 27 Nov 2019, median exposure duration: 8.6 months) or that of 29 patients with HR-MDS or AML who received MBG453 plus azacitidine (Aza) (cutoff 14 Jan 2020, median exposure duration: 3.0 months) was 58% and 70% for HR-MDS, respectively, and 41% and 27%, respectively, for newly diagnosed (ND)-AML (NCT03066648) (88). Moreover, the ORR was only 24% for MBG453 plus Dec in patients with R/R AML (**Supplementary Table 1**). The most common grade 3/4 treatment-emergent (TE) adverse events (AEs) (in Dec and Aza group) were thrombocytopenia, febrile neutropenia, neutropenia, and anemia. For MBG453 plus Dec, only 4 patients experienced potential immune-related (IR) AEs that were reported as treatment-related (ALT increase, arthritis, hepatitis, hypothyroidism, rash). No treatment-related ≥ grade 3 potential IRAEs have been reported for MBG453+Aza (**Supplementary Table 2**). No treatment-related grade 4 IRAEs or deaths had been reported for either combination. Overall, MBG453 plus Dec or Aza is safe and well tolerated in HR-MDS and AML, which showed encouraging response rates and emerging durability as well (88, 90). However, whether TIM-3 inhibitors together with HMA treatment could improve clinical outcome requires more evidence from these trials. STIMULUS-MDS1 (N≈120; NCT03946670) is a double-blind, phase II clinical trial to evaluate whether MBG453 plus HMAs improves the CR rate and progression-free survival (PFS) *vs*. HMA alone in HR-MDS (89). STIMULUS-MDS2 (N≈500, NCT04266301) is a phase III clinical trial to further confirm whether MBG453 prolongs the OS of HR-MDS patients including CMML-2, and similar trials are ongoing in China and are recruiting (CTR20201781). STIMULUS-AML (N≈86; NCT04150029) is an open-label study evaluating the safety (DLTs) and efficacy (CR rate) of MBG453+HMA combined with venetoclax in ND-AML (89). In addition, ongoing clinical studies included the application of MBG453 in MRD-positive patients after allo-HSCT (NCT04623216) and in combination with a PD-1 inhibitor (NCT0306664), TP53-MDM2 inhibitor (NCT03940352), or Bcl-2 inhibitors plus HMAs (NCT04812548, NCT04150029) for efficacy and safety in MDS and AML. Similarly, a phase I clinical study of the efficacy and safety of another TIM-3 inhibitor, SHR-1702, for the treatment of R/R-AML and HR-MDS is ongoing as well (42 cases; NCT04443751) (86). Some studies have shown that TIM-3 inhibitors are well tolerated, and preliminary antitumor activity in advanced solid tumors has been demonstrated for this class of drugs (87, 91). Overall, TIM-3 blockade combined with other checkpoint inhibitors, targeted inhibitors, or HMAs may improve the clinical outcome of patients with leukemia; however, more clinical data are required to support the application of TIM-3 inhibitors in leukemia immunotherapy.

## 5. Concluding Remarks

Numerous clinical studies have indicated that overexpression of TIM-3 is associated with poor prognosis in leukemia. As an immune checkpoint protein, TIM-3 is upregulated on T cells, resulting in increased T cell exhaustion in leukemia patients. TIM-3 is also expressed on leukemia cells and may serve as a biomarker and target for targeted therapy for different leukemias. However, blockade of TIM-3 may lead to different outcomes in AML in comparison with other leukemias. Therefore, further studies and more detailed clinical data are required to evaluate the efficacy of TIM-3 inhibitors in different types and stages of leukemia.

It is necessary to define how TIM-3 affects the function of other immune cells, and if there are other links between TIM-3 overexpression on different immune cells and resistance to different therapies, relapse, and overall survival. Some clinical trials have shown that blockade of TIM-3 alone fails to achieve clinical efficacy for most patients with AML or MDS. It is only when TIM-3 is combined with other checkpoint inhibitors, TKIs, or HMAs that improvements in clinical outcome are observed. Thus, the mechanism of TIM-3 blockade on leukemia cells, in the leukemia bone marrow microenvironment, and on T cells need to be characterized. This research may help to understand how to optimize TIM-3 blockade for leukemia immunotherapy.

## Author Contributions

MGH and MR conceptualized the manuscript. CZ and YL finalized the manuscript. JT and MR generated the figures. All authors contributed to the article and approved the submitted version.

## Funding

This study was supported by grant from the National Natural Science Foundation of China (No. 82070152).

## Conflict of Interest

The authors declare that the research was conducted in the absence of any commercial or financial relationships that could be construed as a potential conflict of interest.

## Publisher’s Note

All claims expressed in this article are solely those of the authors and do not necessarily represent those of their affiliated organizations, or those of the publisher, the editors and the reviewers. Any product that may be evaluated in this article, or claim that may be made by its manufacturer, is not guaranteed or endorsed by the publisher.
