# Supplementary material for: TIM-3 in Leukemia; Immune Response and Beyond
Source: Front Oncol. 2021 Sep 30;11:753677. doi: 10.3389/fonc.2021.753677 (PMC8514831; doi:10.3389/fonc.2021.753677)
Supplement: Supplementary file 1 [file Table_1.docx]

Supplementary Table 1.

The ORR of MBG453 plus HMA in HR-MDS and AML

| Disease | Intervention | ORR |
| --- | --- | --- |
| HR-MDS | MBG453+Dec | 58% |
|  | MBG453+Aza | 70% |
| ND-AML | MBG453+Dec | 41% |
|  | MBG453+Aza | 27% |
| R/R-AML | MBG453+Dec | 24% |

**Abbreviations:** AML, acute myeloid leukemia; Aza, Azacitidine; Dec, Decitabine; HR-MDS, higher-risk myelodysplastic syndromes; HMA, hypomethylating agents; ND, newly diagnosed; ORR, overall response rate; R/R, relapsed/refractory.

Supplementary Table 2.

Incidence of AEs of MBG453 plus HMA in HR-MDS and AML

| Intervention | TEAEs (%) | | | | IRAEs |
| --- | --- | --- | --- | --- | --- |
|  | thrombocytopenia | febrile neutropenia | neutropenia | anemia |  |
| MBG453+Dec | 41% | 46% | 42% | 25% | 6% |
| MBG453+Aza | 52% | 21% | 38% | 28% | 0 |

**Abbreviations:** AEs, adverse events; Aza, Azacitidine; Dec, Decitabine; TEAEs, treatment-emergent adverse events; IRAEs, immune-related adverse events.
